# Ribosome traffic jam in neurodegeneration: decoding hurdles in Huntington disease

**DOI:** 10.15698/cst2021.06.251

**Published:** 2021-05-03

**Authors:** Srinivasa Subramaniam

**Affiliations:** 1 The Scripps Research Institute, Department of Neuroscience, Jupiter, FL, USA.

**Keywords:** jamming, translocation, codon pause, striatum, brain disorder, mitochondria, occupancy

## Abstract

A ribosome typically moves at a particular rate on a given mRNA transcript to decode the nucleic acid information required to synthesize proteins. The speed and directionality of the ribosome movements during mRNA translation are determined by the mRNA sequence and structure and by various decoding factors. However, the molecular mechanisms of this remarkable movement during protein synthesis, or its relevance in brain disorders, remain unknown. Recent studies have indicated that defects in protein synthesis occur in various neurodegenerative diseases, but the mechanistic details are unclear. This is a major problem because identifying the factors that determine protein synthesis defects may offer new avenues for developing therapeutic remedies for currently incurable diseases like neurodegenerative disorders. Based on our recent study (Eshraghi *et al.*, Nat Commun 12(1):1461; doi: 10.1038/s41467-021-21637-y), this short commentary will review the mechanistic understanding of Huntingtin (HTT)-mediated ribosome stalling indicating that central defects in protein synthesis in Huntington disease (HD) are orchestrated by jamming of ribosomes on mRNA transcripts.

The symptoms of HD, a neurodegenerative disorder, are severe disruption of cognitive and motor functions, including changes in posture and gait. HTT, the gene responsible for HD, is CAG (codes for glutamine (Q)) expanded and encodes a 350-kd protein (**Figure 1**) that has a role in neuronal survival, vesicular trafficking, and transcription, as well as in immune cell functions. However, the mechanism by which poly-Q expanded mutant HTT (mHTT) promotes toxicity remains enigmatic, and its complexity has precluded the development of effective HD drugs. This situation is further complicated by the fact that mHTT is ubiquitously expressed and yet it promotes massive neuronal degeneration in the striatum that then produces peripheral defects, such as skeletal muscle atrophy.

**Figure 1:**
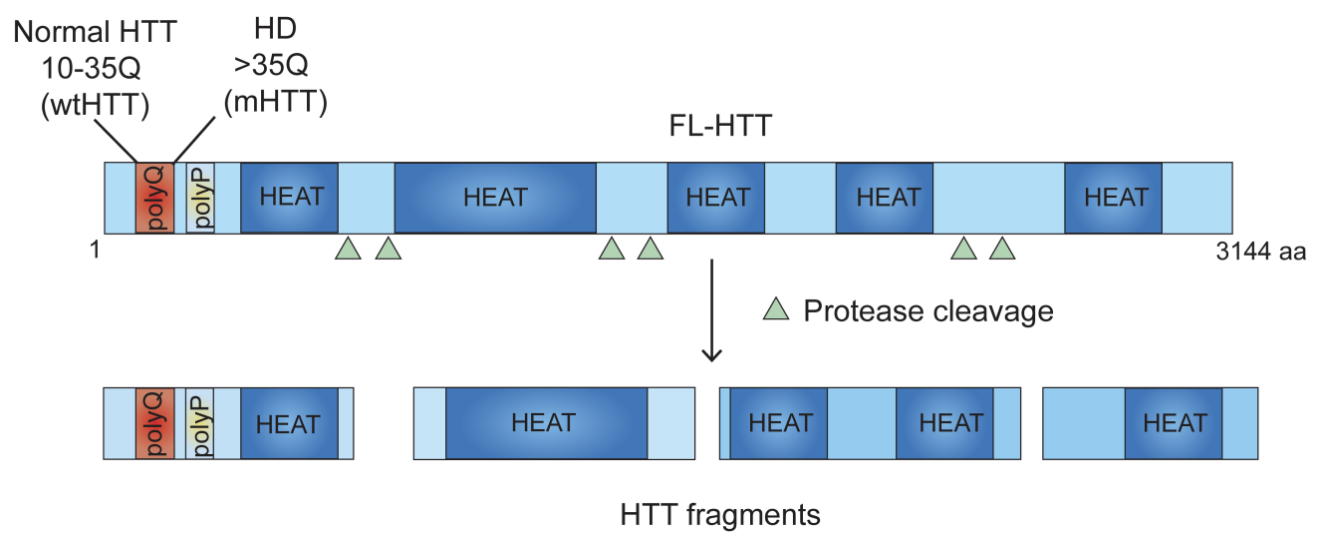
FIGURE 1: Domain structure of HTT protein. HTT consists of 3144 amino acids (aa). PolyQ-polyglutamine tract; polyP-proline-rich domain; HEAT repeats are shown. polyQ and polyP may regulate protein-protein interaction. HEAT repeats may act as scaffolding domain for tethering, localizing, and coordinating the functions of the proteins involved in ribosome elongation, thereby controlling the movement of ribosomes. polyQ expansion in HTT (mHTT) inhibits the movement of ribosomes causing stalling of the polyribosomes. HTT undergoes proteolytic cleavage and generates HTT fragments that may also regulate ribosome movements. Triangle depicts approximate location of protease cleavage sites.

A new mechanism of HTT action in regulating ribosome movement is revealed in our recent report by Eshraghi *et al.*, in which we show that both wildtype HTT (wtHTT) and mHTT can bind to ribosomes and slow down ribosome movement; however, mHTT binds much stronger. We used a combination of biochemical and sequencing methods to show that wtHTT inhibits ribosomal movements and that this function is further enhanced by mHTT. The end result is that mHTT causes ribosomal jamming and a consequent decrease in protein synthesis (**Figure 2A-C**).

**Figure 2:**
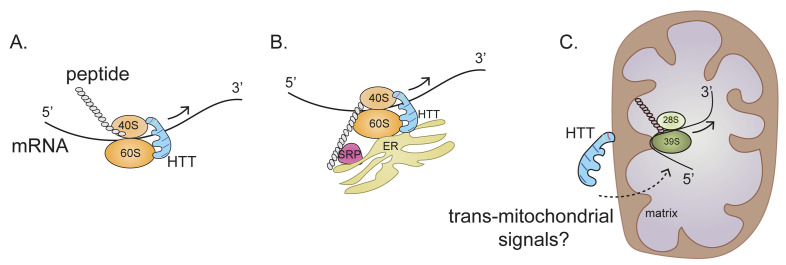
FIGURE 2: HTT (wtHTT and mHTT) binds to 40S and 60S ribosomal subunits and regulates the ribosome movement by acting as “molecular brake” thereby altering the ribosomal occupancy of selected cytoplasmic mRNAs (A), ER-associated mRNAs (B), and mitochondrially-encoded OXPHOS mRNAs (C). HTT is depicted as scaffolding protein. Arrow indicates the direction of ribosomal movement. Dotted arrow indicates potential signals through which HTT may. regulate mitochondrial mRNAs ribosome translocation. See text for details.

A role for mHTT in protein synthesis has been recognized for some time, but the molecular mechanisms of how mHTT blocks protein synthesis are still unclear. Our ribosome-sequencing (Ribo-Seq) data suggest that translating ribosomes are differentially accumulated on several hundred mRNAs in HD cells. The Ribo-Seq approach provides a “snapshot” of all the ribosomes active in a cell at a specific time point, so it preserves the ribosome location during translation and documents the ribosome position in the 5' to 3' direction on mRNA. We found an interesting accumulation of ribosomes at the 5' position on the mRNAs of HD cells that expressed both copies (homozygous) of mHTT, but a 3' accumulation of ribosomes on mRNAs of HD cells expressing one copy (heterozygous) of mHTT. No evidence presently indicates a particular “signature” of mRNAs whose ribosome occupancy is altered in HD; however, this differential ribosome occupancy indicates that the amount of mHTT will determine the ribosome distribution across the various mRNA types. This most likely reflects differential ribosome binding of wtHTT versus mHTT.

Any mechanism proposed to explain how HTT promotes this ribosome stalling must take into account the complexity of mRNA translation and the various intracellular locations where translation occurs. Approximately half of the mRNAs that encode cytosolic proteins and almost all mRNAs encoding secretory and membrane proteins are bound to the endoplasmic reticulum (ER). Thus, the ER is now emerging as a major site for the translation of mRNAs that encode both cytosolic and targeted proteins. HTT may bind to 40S and 60S ribosomal subunits in the cytosol or in the ER and then regulate ribosome translocation on specific mRNAs in a compartment-dependent manner (**Figure 2A, B**). HTT may also control ribosomal translocation by differential binding to the 5' and 3' ends of mRNAs and to other non-ribosomal proteins such as signal recognition particle (SRP) involved in translation elongation. Thus, the main role of HTT throughout the elongation process may be to act as a scaffold that facilitates ribosomal movement, thereby functioning as a “molecular brake” (**Figure 2**). Impeded elongation may also promote the formation of a stalled initiation complex downstream comprising ribonucleoprotein components known as stress granules (SGs). Thus, HTT, which interacts with caprin1, a SG marker, may be recruited to SGs to diminish protein synthesis also by blocking the initiation of translation.

Many aspects of these processes are unknown. The existence of HTT fragments (**Figure 1**) suggests that both the full-length and fragmented forms may have inhibitory effects by serving as scaffolds for interacting with ribosomes. The HTT region involved in the regulation of ribosome movements is presently unidentified. HTT domain analysis reveals an unusual occurrence of 28–36 Huntington/Elongation/A subunit/Target-of-rapamycin (HEAT) repeats with a conserved amino acid sequence that is often found in protein synthesis assemblies, such as eIF4G, that bind to particular RNA- and protein-binding motifs. We predict that many of these HEAT repeat sites in HTT may be regulating ribosome movements; however, further structural analysis is necessary to identify the locations of specific ribosomal proteins and the positions of defined segments of rRNA, mRNA, or protein ligands associated with HTT. Therefore, structural determination studies (e.g., using cryo-EM of HTT with ribosomal subunits) will substantially improve our understanding of how HTT modulates ribosome movements.

Our Ribo-Seq data has revealed a central perturbance of ribosome occupancy of mitochondrially encoded mitochondrial OXSPHOS mRNA in HD cells (**Figure 2C**). This effect is so distinctive that almost no alterations in ribosome occupancy were detected in nuclear-encoded OXPHOS mRNA in HD cells. Perhaps the most challenging question is the nature of the mechanisms used by mHTT for selective regulation of ribosome occupancy of mitochondrially encoded mRNA, as this translation takes place inside the mitochondrion (**Figure 2C**). The implied involvement of mHTT in OXPHOS ribosome occupancy is conceptually problematic because, unlike the case for cytoplasmic mRNAs, no clear evidence indicates a localization of mHTT inside the mitochondrial matrix. One tantalizing possibility is that association of mHTT with the mitochondrial membrane can elicit signals that trigger ribosome occupancy in the matrix, so that mHTT also regulates mitochondrial protein synthesis via trans-mitochondrial signaling across the membrane (**Figure 2C**).

Ribosomal occupancy on mRNAs is not uniform, and this reflects its regulatory roles. Ribosomal pauses may modify the rate of protein synthesis, co-translational folding, modifications, and destination events, thereby necessitating ribosomal pausing as a major driver of controlled protein synthesis. In HD, ribosome pauses can manifest detrimental effects by differential expression of selected mRNAs that might contribute to disease pathology. Enhanced pauses may elicit ribosomal stalling, followed by deleterious collisions and diminished protein synthesis. What remains unclear is whether all ribosomal pauses in HD lead to diminished protein synthesis. This scenario is unlikely, as *Fmr1* showed a higher ribosome occupancy but enhanced protein synthesis, whereas *Mfsd10* showed a higher ribosome occupancy but diminished protein levels, indicating that additional mechanisms contribute to protein-level regulation in HD. Nevertheless, the establishment of differential ribosome pauses on mRNA may serve as a primary step for stable decoding of mRNA and proper functioning of newly formed proteins.

Much of the understanding of the molecular basis of ribosomal pauses comes from studies in non-mammalian systems, such as yeast and bacteria. The basics of ribosome structure and protein synthesis have been conserved evolutionarily to a remarkable extent, but whether similar molecular mechanisms are used by dauntingly complex mammalian cells such as neurons, with their intra-neuronal and inter-neuronal communication, is not known. Research is now beginning to unravel the molecular mechanism and role of ribosomal pauses in mammalian systems. Obtaining a complete understanding of how ribosome stalling affects HD onset and disease progression will take many years; however, our complete Ribo-Seq database offers a valuable foundation for identifying and ascertaining the roles of novel regulator(s) that are translationally dysregulated and potentially contribute to HD.

These new findings provide examples of how mHTT may affect HD progression by impeding ribosomal movements. This then disrupts systematic decoding of mRNA and generates mismatches, misfolded proteins, and aberrant translational products. Rigorous mechanistic studies could therefore reveal new insights into the roles of ribosome stalling and mRNA translational dysregulation in the neuronal vulnerability seen in neurodegenerative disorders.

